# Low Levels of Human HIP14 Are Sufficient to Rescue Neuropathological, Behavioural, and Enzymatic Defects Due to Loss of Murine HIP14 in *Hip14*−/− Mice

**DOI:** 10.1371/journal.pone.0036315

**Published:** 2012-05-23

**Authors:** Fiona B. Young, Sonia Franciosi, Amanda Spreeuw, Yu Deng, Shaun Sanders, Natalie C. M. Tam, Kun Huang, Roshni R. Singaraja, Weining Zhang, Nagat Bissada, Chris Kay, Michael R. Hayden

**Affiliations:** Department of Medical Genetics and Centre for Molecular Medicine and Therapeutics, Child and Family Research Institute, University of British Columbia, Vancouver, British Columbia, Canada; Emory University, United States of America

## Abstract

Huntingtin Interacting Protein 14 (HIP14) is a palmitoyl acyl transferase (PAT) that was first identified due to altered interaction with mutant huntingtin, the protein responsible for Huntington Disease (HD). HIP14 palmitoylates a specific set of neuronal substrates critical at the synapse, and downregulation of HIP14 by siRNA *in vitro* results in increased cell death in neurons. We previously reported that mice lacking murine *Hip14* (*Hip14−/−*) share features of HD. In the current study, we have generated human *HIP14* BAC transgenic mice and crossed them to the *Hip14−/−* model in order to confirm that the defects seen in *Hip14−/−* mice are in fact due to loss of *Hip14*. In addition, we sought to determine whether human *HIP14* can provide functional compensation for loss of murine *Hip14*. We demonstrate that despite a relative low level of expression, as assessed via Western blot, BAC-derived human *HIP14* compensates for deficits in neuropathology, behavior, and PAT enzyme function seen in the *Hip14−/−* model. Our findings yield important insights into HIP14 function *in vivo*.

## Introduction

Protein palmitoylation involves the reversible addition of palmitic acid to cysteine residues via a thioester bond, and plays a key role in protein trafficking and neuronal function [1], [2]. Twenty-three mammalian palmitoyl-acyl transferases (PATs), each bearing a characteristic DHHC domain [3] and displaying distinct substrate specificity [4] were identified as the enzymes responsible for catalyzing protein S-palmitoylation. Palmitoylation plays a critical role in neuronal function and health [5], [6]. For example, PAT dysfunction or loss has been associated with several human diseases, including mental retardation (OMIM *300646, *300576) [7], [8], schizophrenia (OMIM *608784) [9]–[11], and Alzheimer's Disease (#104300) [12]. In contrast, far less is known about the acyl protein thioesterases that are thought to catalyze palmitate removal [13], but their importance in regulation of protein palmitoylation has been highlighted by patients with mutations in the thioesterase PPT1 (Entrez Gene ID 5538) that result in neuronal ceroid lipofuscinosis [14].

Huntington Disease (HD, OMIM #143100) is an autosomal dominant neurodegenerative disease that presents with cognitive, motor, and psychiatric signs and symptoms [15]. Striatal volume loss due to medium spiny neuron (MSN) degeneration is a key feature of the disease [16]. HD results from an expansion of the CAG repeat in the *HD* gene, resulting in a polyglutamine (poly-Q) expansion in the N-terminus of the huntingtin (HTT) protein [17]. Huntingtin Interacting Protein 14 (HIP14, Entrez Gene ID 23390), also known as DHHC17, was first identified as part of a yeast-two-hybrid screen for proteins that interact with HTT (Entrez Gene ID 3064) [18]. Sequence similarity of HIP14 to Akr1p (Entrez Gene ID 851857; one of the first reported PATs and a protein required for endocytosis) together with the ability of human HIP14 to rescue Akr1p trafficking defects, led to the formal description of HIP14 as the first mammalian PAT soon after [19].

Many widely divergent proteins interact with HTT [20]. However, HIP14 was selected for further study because its interaction with HTT is reduced in the presence of the mutation responsible for HD [18], resulting in less robust palmitoylation of HIP14 substrates [21] fulfilling genetic criteria for having a potential relationship to the disease. The enrichment of HIP14 in the brain, its expression in the medium spiny neurons primarily affected in HD, and its co-localization with HTT are all features supportive of a role for HIP14 in the pathogenesis of HD [18]. HIP14 demonstrates PAT substrate specificity for many neuronal proteins, including HTT as well as PSD-95 (Entrez Gene ID 1742), SNAP-25 (Entrez Gene ID 6616), and NR2B (Entrez Gene ID 2904) [19]. More recently, the major site of palmitoylation of HTT was identified as cysteine 214 and mutation of this site, rendering HTT non-palmitoylatable, increases inclusion formation and neuronal toxicity. Similar results are obtained by treating cells with *HIP14* siRNA, whereas overexpression of *HIP14* reduces the appearance of inclusions [22].

**Table 1:** Sequences of primers used in this study.

	Primer name	Description		Application	Primer sequence
**1**	HIP14h_Pr1	Upstream/promoter area 1	F	PCR genotyping	cccagaggtccaaacaacat
			R		gcttttccaaccaggcttc
**2**	HIP14h_Pr2	Upstream/promoter area 2	F	PCR genotyping	tatttccctgcttccaatgc
			R		ttccccacttcctgtctctg
**3**	HIP14h_Ex1	Exon 1	F	PCR genotyping	cgggaggagggatttaacac
			R		gagtccggggaagaaagg
**4**	HIP14h_Int1	Intron 1	F	PCR genotyping	gaaccgtgctgagtggattc
			R		acacctccacctctgtcctc
**5**	Ex_7	Exon 7	F	PCR genotyping	cacagtcattagccttcttctgg
			R		gggtcctcctatcaacaccat
**6**	STOP	Stop codon (spans stop codon)	F	PCR genotyping	tttggggtgctgtttttagc
			R		attttcaggcaccactcagc
**7**	3′UTR	3′ untranslated region	F	PCR genotyping	aatgggcgtaaaacagcatc
			R		ccacagaataacacggtaagtagc
**8**	hHIP14 F2/R2	Exon 1	F	qPCR	ccccgggagggtgaaac
			R		agcccgcttcggtatcgta
**9**	hHIP14 F1/R1	Exon 1–2 (intron-spanning)	F	qRT-PCR	taccgaagcgggctgtgt
			R		agttttccgtccaagaggttcac
**10**	mHIP14 rt2f_F1/R1	Exon 14–15 (intron spanning)	F	qRT-PCR	tggttgtgtctcttactgggg
			R		catccacggggagcatgtg
**11**	mbact F1/R1	mouse actin (endogenous control)	F	qPCR & qRT-PCR	acggccaggtcatcactattg
			R		caagaaggaaggctggaaaaga

**Figure 1:**
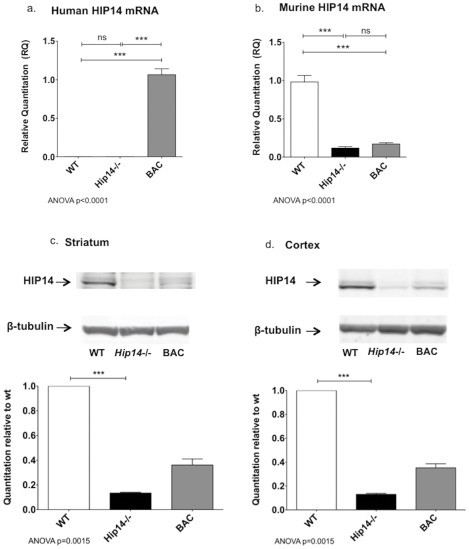
HIP14 mRNA and protein expression in *Hip14−/−* and *HIP14* BAC mice. Human *HIP14* mRNA (**a**) is expressed in BAC mice (p<0.0001) and no signal is detected in WT or *Hip14*−/− littermates (ANOVA p<0.0001, n = 6). Murine *Hip14* mRNA transcript (**b**) is significantly reduced in *Hip14−/−* mice (p<0.0001) and is not altered in the presence of human *HIP14* (ANOVA p<0.0001, n = 6). Primers for mouse actin were used as an endogenous control. HIP14 protein expression in striatum (**c**) and cortex (**d**) was assessed by western blot using an in-house HIP14 polyclonal antibody. Beta-tubulin was used as a loading control. Striatum and cortex both n = 5. Post-hoc Tukey tests: ***p<0.0001.

Evidence supporting a protective role for HIP14 and further implicating HIP14 in the pathogenesis of HD was obtained through generation and characterization of a mouse lacking murine *Hip14* (*Hip14−/−*, Entrez Gene ID 320150) [23]. These mice bear features similar to those seen in the YAC128 mouse model of HD. The latter transgenic model is widely studied and recapitulates many features of human HD, including loss of MSNs and striatal volume with accompanying motor, cognitive, and affective dysfunction [24]–[26]. However, these phenotypes appear earlier in the *Hip14−/−* mouse and are of greater severity, in addition to being non-progressive [23]. For example, the *Hip14−/−* mouse displays a 17% loss in striatal volume by embryonic day E17.5, as compared to 9 months in the YAC128 mouse [24]. In addition, the *Hip14−/−* mice demonstrate deficits in motor function and palmitoylation of HIP14 substrates, again both features observed in the YAC128 model.

**Table 2:** A table summarizing the genotyping results for the 11 founders.

Primer	Primer 1	Primer 2	Primer 3	Primer 4	Primer 5	Primer 6	Primer 7
Region	Promoter region 1	Promoter region 2	Exon 1	Intron 1	Exon 7	STOP	3′ UTR
Founder line							
HB1	+	+	+	+	+	+	+
HB2	+	+	+	+	+	+	+
HB3	+	+	+	+	+	+	+
HB4	+	+	+	+	+	+	+
HB5	+	+	+	+	+	+	+
HB6	+	+	+	+	+	+	+
HB7	+	+	+	+	+	+	+
HB8	+	+	−	−	−	−	−
HB9	+	+	+	+	+	+	+
HB10	+	+	+	−	−	−	−
HB11	+	+	+	+	+	+	+

A “+" sign indicates that a PCR product was detected. A “−" sign indicates that no PCR product was detected, suggesting that a truncated BAC construct was integrated in these lines.

Due to the putative protective role for HIP14 in HD suggested by features of HD observed in the *Hip14−/−* mice, we sought to create a mouse that overexpresses HIP14 in order to obtain a greater understanding of HIP14 biology *in vivo*. To help ensure a pattern of HIP14 overexpression closely resembling that of normal physiology, we selected a Bacterial Artificial Chromosome (BAC) transgenic approach, which permits inclusion of endogenous regulatory DNA surrounding the gene of interest [27], [28]. Because our primary goal remains to understand HIP14 biology as it pertains to human disease, we selected a human *HIP14* transgene.

**Figure 2:**
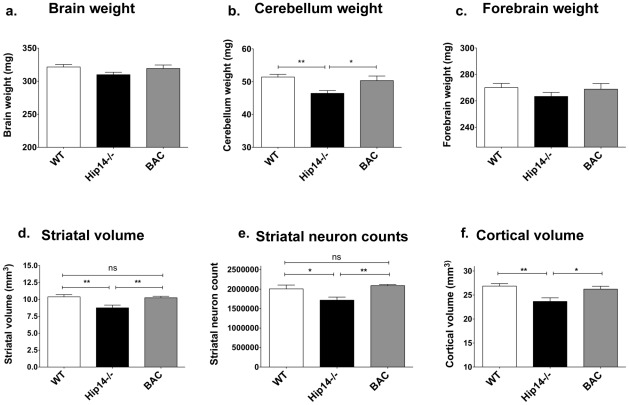
HIP14 rescues the neuropathological deficits seen in the *Hip14*−/− mouse. Mice aged 1 month were perfused with 4% PFA and sectioned using a cryostat, stained with anti-NeuN, mounted and volumes and neuronal counts were measured using Stereo investigator. **a.** Total brain weight shows a trend to rescue in mice aged 1 month, although one-way ANOVA analysis is not significant (WT: 321.5±3.78, *Hip14−/−:* 309.8±3.74, BAC: 319.2±5.32 g, ANOVA p = 0.14). Pairwise t-tests reveal that *Hip14−/−* whole brain weight is significantly decreased compared to WT (p = 0.04) and that BAC do not differ from WT (p = 0.7). **b.** Cerebellum weight is significantly decreased in *Hip14*−/− mice and is rescued to WT levels in BAC mice at 1 month (WT: 51.41±0.82, *Hip14−/−:* 46.45±0.83, BAC: 50.34±1.38 g, ANOVA p = 0.0038). Pairwise t-test analysis similarly reveals that *Hip14−/−* cerebellum is significantly decreased compared to WT (p = 0.0003) and that BAC do not differ from WT (p = 0.5). **c.** Forebrain weight shows a similar trend to rescue at 1 month of age (WT: 270.1±3.15, *Hip14−/−:* 263.4±3.06, BAC: 268.9±4.27 g, ANOVA p = 0.36). **d.** Striatal volume is significantly rescued in BAC mice at 1 month of age (WT:10.4±0.3, *Hip14*−/−: 8.8±0.4 mm^3^, BAC: 10.3±0.2 mm^3^; ANOVA p = 0.002). **e.** Striatal neuron count is likewise rescued by the human *HIP14* BAC at 1 month of age (WT:2.0±0.1, *Hip14*−/−: 1.7±0.08, BAC: 2.1±0.03 million cells; ANOVA p = 0.004). **f.** Finally, cortical volume loss observed in *Hip14−/−* mice is also rescued by human HIP14 at 1 month (WT:26.86±0.52, *Hip14*−/−: 23.66±0.75, BAC: 26.22±0.60 mm^3^; ANOVA p = 0.003). Similar observations are made at 3 months of age (data not shown). 1 month n = 12, 12, and 11 for WT, *Hip14*−/−, and BAC respectively. * p<0.05, **p<0.01, ***p<0.0001.

The advantages of using the human gene when generating transgenic mice in relation to the study of human disease has been demonstrated in mouse models where the human gene is used in artificial chromosome systems of transgenesis [29], [30]. These studies have been highly successful in generating mice that accurately recapitulate the key aspects of the disease phenotype and likely the underlying molecular cause of disease in patients, rendering these models suitable for future use in preclinical studies. Ultimately, a mouse overexpressing human HIP14 may be crossed to mouse models of HD, anticipating that HIP14 overexpression might delay the onset of the features of HD, or reduce their severity.

**Figure 3:**
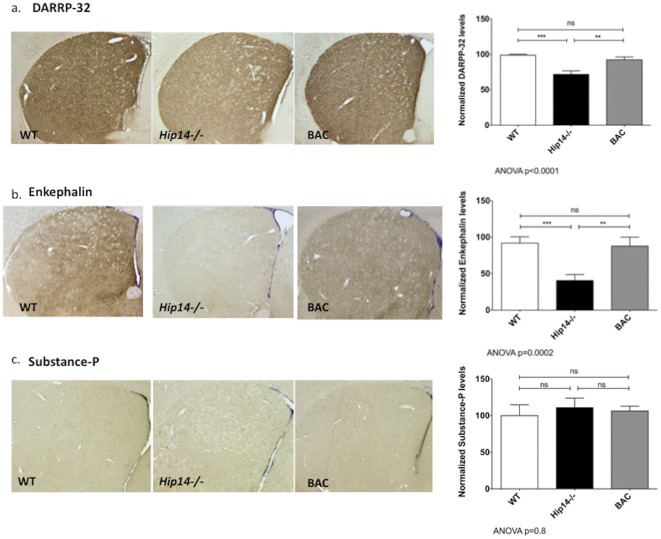
BAC-derived HIP14 rescues the neurochemical phenotype seen in *Hip14*−/− mice. Immunohistochemistry was assessed in mice aged 1 month. Staining intensity was reduced in the *Hip14−/−* and restored to normal levels for **a.** DARRP-32 (WT: 98.8±1.4, *Hip14−/−*: 71.6±4.9, BAC: 92.4±3.9; ANOVA p<0.0001) and **b.** Enkephalin (WT: 91.9±8.5, *Hip14−/−*: 40.5±8.2, BAC: 87.7±12.3; ANOVA p = 0.0002). **c.** Substance-P was unchanged in all three genotypes (WT: 100.0±14.8, *Hip14−/−*: 110.8±13.0, BAC: 106.4±6.4; ANOVA p = 0.8)***p<0.0001, **p<0.01.

**Figure 4:**
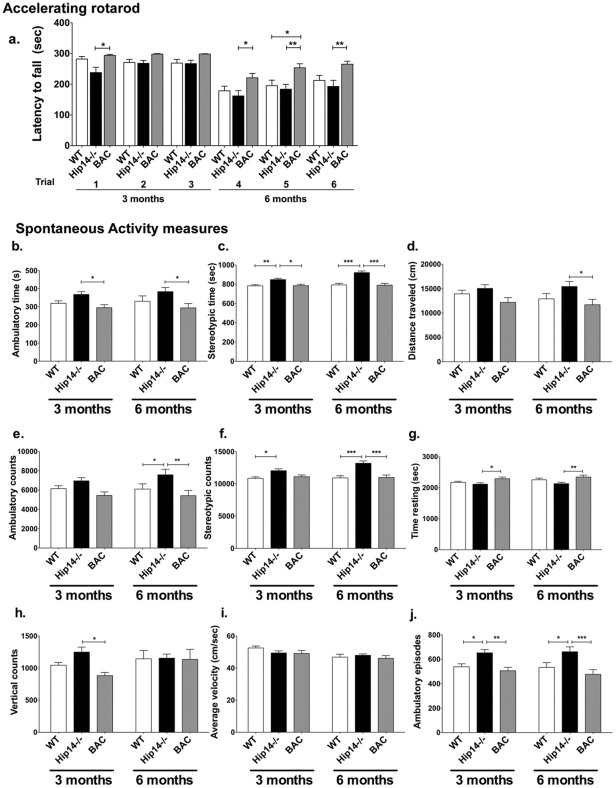
Human HIP14 rescues behavioural alterations in the *Hip14−/−* mouse. Deficits in motor coordination and balance are rescued by BAC-derived human HIP14. **a.** Repeated measures ANOVA in combined genders reveals a significant effect of genotype F(2,300) = 600, p = 0.0035. Bonferroni post-hoc analysis reveals that BAC mice perform consistently better on the accelerating rotarod task than *Hip14*−/− mice (p<0.05 in trials 1,4; p<0.01 in trials 5,6) and superior to WT mice in later trials (p<0.05 trial 5). All other post-hoc comparisons are non-significant (p>0.05). n = 25, 20, and 17 for WT, *Hip14*−/−, and BAC respectively. (**b–j**) Human HIP14 normalizes the hyperactivity observed in *Hip14*−/− mice to WT levels. Spontaneous activity was assessed in Med Associates boxes at 3 and 6 months of age. Repeated measures ANOVA reveals a significant effect of genotype in ambulatory time (**b;** F(2,53) = 5.05, p = 0.0099) and counts (**e;** F(2,53) = 5.36, p = 0.0076), stereotypic time (**c**; F(2,53) = 19.70, p<0.0001) and counts (**f**; F(2,53) = 15.13, p<0.0001), distance traveled **(d;** F(2,53) = 3.60, p = 0.034), time resting (**g;**(2,53) = 4.99, p = 0.010), and ambulatory episodes (**j**; F(2,53) = 8.68, p = 0.0005). A significant effect was not seen in vertical counts **(h;** F(2,51) = 1.89, p = 0.1611**)**, and average velocity **(i;** F(2,54) = 0.68, p = 0.51**)**. The most robust effects were observed for stereotypic time (**c**) and counts (**f**). Single-housed mice were excluded from all analyses.*p<0.05, **p<0.01, ***p<0.0001.

Because of the very high level of sequence conservation between human and mouse HIP14 protein (98% identical), we predicted that human *HIP14* would be compatible with the murine cellular and transcriptional machinery. Previous studies demonstrate that many human proteins can fully [29], [31] or partially [32]–[35] rescue the murine null phenotype.

**Figure 5:**
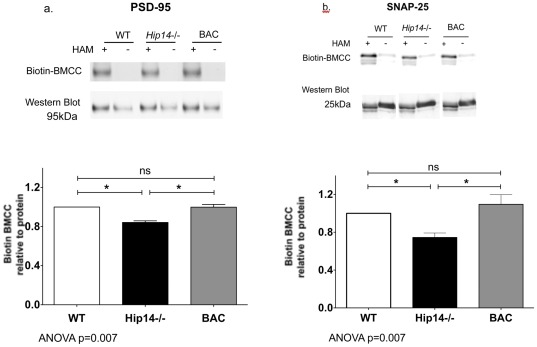
Rescue of palmitoylation of key HIP14 in substrates in the *Hip14*−/− mouse. Rescue of palmitoylation deficits of key HIP14 substrates in the *Hip14*−/− mouse, as assessed by Biotin BMCC assay on HIP14 substrates PSD-95 (a) and SNAP-25 (b). ANOVA p = −0.007, n = 5 each. *p<0.05.

The objective of this study was to confirm that defects seen in *Hip14−/−* mice are indeed the result of the absence of HIP14. In addition, we sought to determine the levels of HIP14 sufficient to rescue the phenotype in *Hip14−/−* mice and whether certain endpoints are more sensitive to loss of murine *Hip14*. Finally, we wanted to address whether human HIP14 can compensate for loss of the murine protein.

**Figure 6:**
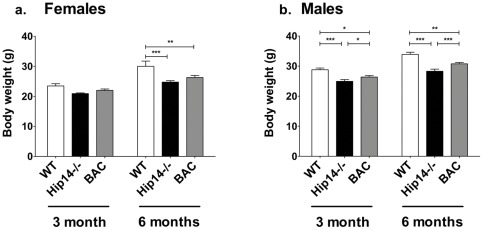
BAC-derived Human HIP14 is not sufficient to rescue body weight in the *Hip14*−/− mice. *Hip14−/−* mice demonstrate reduced body weight as early as 2 weeks of age (data not shown). **a.** Body weight is decreased in *Hip14−/−* females (89.2% of WT) at 3 months of age, and this is partially rescued (93.8% of WT) in BAC mice (WT 23.47±0.76, *Hip14*−/− 20.94±0.23 g, BAC 22.02±0.44 g). At 6 months, a similar partial rescue is observed (WT 30.00±1.74, *Hip14*−/− 24.75±0.49 g and 82.5% of WT, BAC 26.29±0.69 g and 87.6% of WT). Repeated measures ANOVA reveals a significant effect of genotype in females alone (F(2,27) = 7.53, p = 0.0025). Bonferroni post-tests reveal that WT differs significantly from both *Hip14*−/− (p<0.0001) and BAC (p<0.01). n = 10, 10, and 9 for WT, *Hip14*−/−, and BAC respectively. **b.** In males, *Hip14−/−* body weight is similarly decreased at 3 months (86.7% of WT) with partial rescue (91.7% of WT) in BAC (WT 28.79±0.56, *Hip14*−/− 24.95±0.57 g, BAC 26.39±0.47 g). By 6 months, partial rescue is further apparent (WT 33.91±0.71, *Hip14*−/− 28.30±0.70 g and 83.45% of WT, BAC 30.78±0.44 g and 90.75% of WT). A highly significant effect of genotype is present in males (F(2,37) = 26.81, p<0.0001). Bonferroni post-tests reveal a highly significant difference between WT and *Hip14*−/− at 3 and 6 months of age (p<0.0001), and a significant, but less robust, difference between WT and BAC mice at both ages (3 months p<0.05, 6 months p<0.01). However, BAC mice are also significantly different from *Hip14−/−* at both 3 (p<0.05) and 6 months (p<0.0001). n = 15, 10, and 8 for WT, *Hip14*−/−, and BAC respectively.

In this study, we have generated a human *HIP14* BAC transgenic mouse and confirmed a functional rescue of the neuropathological, behavioral, and enzymatic deficits observed in the *Hip14−/−* mouse by the human *HIP14* transgene. In this humanized mouse model, we report that human HIP14 compensates for the key features resulting from loss of the murine ortholog.

## Materials and Methods

All experiments were carried out in accordance with protocols (Animal protocols A07-0106 and A07-0262) approved by the UBC Committee on Animal Care and the Canadian Council on Animal Care.

### Generation of *HIP14* BAC mice & animal breeding strategy

A ∼190 kb long human Bacterial Artificial Chromosome (BAC) containing the entire *HIP14* gene (RP11-463M12) was obtained from the Children's Hospital Oakland Research Institute (CHORI) (Figure S1a).

BAC DNA was prepared according to a standard protocol obtained from the Rockefeller University NINDS GENSAT BAC Transgenic project (http://www.gensat.org/GensatProtocols.pdf), microinjected into FVB/N fertilized embryos, and implanted in pseudopregnant female mice. Seven human-specific primers spanning the *HIP14* gene and surrounding sequence were designed in order to characterize founders carrying the entire construct, and for subsequent routine PCR genotyping (Table 1).

### Animal breeding strategy

BAC founders were identified using a standard genotyping protocol. The highest-expressing line (HB4) was chosen for further study, and BAC,*Hip14+/−*×*HIP14+/−* matings were set up to obtain mice for use in the study, namely wildtype (WT) (*Hip14+/+*), *Hip14−/− (Hip14−/−*), and the humanized BAC mice (BAC,*Hip14−/−*).

Mice were genotyped at wean using tail clips, with two PCR reactions: One for the human *HIP14* BAC (Table 1, primer pair 5), and one for mouse *Hip14* as described previously [23]. Genotypes of mice were re-confirmed at time of sacrifice.

### Transgene copy number assessment by quantitative PCR

A primer pair specific for human *HIP14* exon 1 (Table 1, primer pair 8) was designed using Primer Express 3.0 software (ABI). Relative transgene copy number was assessed on the ABI 7500 Fast system using Power SYBR Green PCR Mastermix (Applied Biosystems) and the relative quantitation settings. Signal was normalized to mouse actin (Table 1, primer pair 11).

### Isolation of RNA & quantitative RT-PCR

Total RNA was isolated using the Qiagen RNeasy Plus Mini kit according to the kit instructions. Samples were subsequently treated with DNase I (Invitrogen). cDNA was generated using the SuperScript® III First-Strand Synthesis System (Invitrogen). qRT-PCR was performed on the ABI 7500 Fast Real Time PCR System (Applied Biosystems).

Primers specific for human *HIP14* mRNA (Table 1, primer pair 9) were used in Figure 1a. For Figure 1b, primers specific for murine *Hip14*, annealing 3′ to the gene-trap site in intron 5 were used. Relative gene expression was normalized to mouse actin (as above).

### Preparation of protein lysate and Western Blots

1 month mouse cortex or striatum tissue was homogenized using a Dounce homogenizer on ice in 1 volume of TEEN (50 mM Tris pH 7.5, 1 mM EDTA, 1 mM EGTA, 150 mM NaCl) +1% SDS buffer. After initial lysis, 4 volumes of TEEN +1% Triton X-100 was added. The sample was passed through a 25 gauge needle 5 times and sonicated for 5 seconds on 20% power, spun at 14,000 rpm at 4 degrees for 15 minutes, and the supernatant transferred to a new tube. Protein concentration was determined using the Bradford Assay (Biorad). The above buffers were supplemented with the following reagents: 1× Complete Protease Inhibitors (Roche), 1 mM sodium orthovanadate (Sigma), 800 mM PMSF (Sigma), 5 mM zVAD (Calbiochem). Equal amounts of protein were loaded and run on 4–12% Bis-Tris SDS-PAGE gels (Invitrogen). Immunoblots were obtained using an in-house polyclonal rabbit HIP14 antibody (PEP1) described previously [18]. Beta tubulin antibody T4026 was assessed as a loading control (Sigma).

### Stereology & Neuropathological Assessments

Mice aged 1 month received tail injections of heparin and were terminally anesthetized by intraperitoneal injection of avertin, followed by intracardiac perfusion with fresh cold 4% paraformaldehyde in PBS, pH 7.0 for 10 minutes. Neuropathological assessments were performed as previously described [24]. All analysis was performed with the researcher blind to genotypes.

### Quantification of DARRP-32, Enkephalin and Substance P Immunohistochemistry staining

Immunohistochemistry was performed on 1 month old mice of mixed gender.

Primary antibodies were: mouse anti-DARPP32, #C24-6a (from Dr. P. Greengard and Dr. H. Hemming), rabbit anti-enkephalin (Chemicon), rat anti-substance P (Accurate Chemical). Sections were incubated with horseradish peroxidase (HRP) conjugated secondary antibodies (Jackson ImmunoResearch Laboratories) followed by DAB and 0.5% cresyl violet and visualized using a Zeiss Axioplan 2 microscope. Staining was quantified using MetaMorph (Universal Imaging Corporation). Relative levels of staining were calculated as the sum of the integrated optical density divided by the area selected, then multiplied by the sampling interval (×8) and section thickness (25 µm). The individual values were normalized to wild type.

### Behavioral analysis

Mice were acclimatized to the behavioral testing holding room under reverse lighting (12–10 pm dark cycle) at least one week prior to behavioral testing at 3 months. Group-housed mice of both genders were assessed. Single-housed mice were removed from the analysis, and the experimenter was blind to genotype.

#### Accelerating rotarod test of motor coordination and balance

Motor coordination and balance was assessed as latency to fall on the accelerating rotarod apparatus (Ugo-Basile, Norfolk, UK). Mice were trained for 3 days on a fixed-speed rotarod at 3 months of age. On the fourth day, mice were tested for latency to fall on an accelerating rotarod apparatus as described [25]. Testing was repeated at 6 months of age (without further training).

#### Med Associates spontaneous activity

Spontaneous activity was assessed using the Med Associates activity monitor system (Med Associates Inc., St Albans, VT, USA) as described [23]. Mice were given transgel (Charles River) and acclimatized to the room for at least 1 hour prior to testing, and testing did not commence until 1 hour after the beginning of the dark lighting cycle. The chamber was cleaned with ethanol and allowed to dry between each animal. Each mouse was placed in the center of the testing chamber. Spontaneous activity was recorded for 1 hour.

### Palmitoylation assays

Protein palmitoylation was assessed using the Acyl-Biotin-Exchange with Immunoprecipitation (ABE/IP) method [36] as described previously [23]. For PSD-95, rabbit polyclonal antibody used for IP was generously provided by the late Dr. Alaa El-Husseini. PSD-95 mouse monoclonal antibody MAI-25629 was used for western blot (Affinity Bioreagents, Golden, CO).

For SNAP-25, a mouse monoclonal antibody SMI-81 (Covance, Emeryville, CA) was used for IP, and a rabbit polyclonal SNAP-25 antibody used for western blot (Synaptic Systems #111 002). Palmitoylation (the Biotin-BMCC label) was detected using a Streptavidin Alexa Fluor 680 conjugate antibody (Molecular Probes #S-32358). All palmitoylation assessments were done on whole brain of mice of combined gender aged 1–3 months. Each sample was split in two and processed as technical replicates, in order to reduce assay variability.

### Body weight

Body weight of mice was recorded at 3 & 6 months of age prior to behavioral testing.

### Statistical Analysis

All statistical analyses were performed using the Graphpad Prism software, version 5a. Parametric analysis of single timepoint data was performed using one-way ANOVA, with post-hoc Tukey test. Non-parametric analysis (for western blot and palmitoylation assays) was done using the one-way ANOVA Kruskal-Wallis test with post-hoc analysis using Dunn's Multiple comparison test. Behavioral data collected over multiple time points is assessed using repeated measures ANOVA with Bonferroni post-hoc analysis. Data are reported as mean ± SEM.

## Results

### Generation of *HIP14* BAC mice

A total of 494 FVB/N embyros were injected with a human *HIP14* BAC containing the entire *HIP14* gene, 378 of which survived and were implanted to pseudopregnant females. Forty-five live-born pups were produced, of which 11 were positive for the BAC by PCR genotyping. Nine of these 11 pups produced a PCR product for all primer sets (Figure S1b), indicating integration of an intact BAC construct. Two of eleven lines integrated a partial construct (Table 2).

Assessment of transgene copy number by qPCR in the founders indicated HB4 and HB6 as the two lines with the highest copy number (Figure S1c & d). These were selected for further study, and found to have comparable levels of HIP14 expression (data not shown). Line HB4 was crossed to the existing *Hip14*−/− line in order to asses for rescue in the present study.

### mRNA levels in WT, *Hip14−/−*, and BAC mice

Intron-spanning primers specific for human *HIP14* transcript confirmed the presence of human *HIP14* expression in mice carrying the BAC transgene (BAC). An absence of amplification in the WT or *Hip14−/−* mice (Figure 1a) confirmed that this transcript arises from the human *HIP14* transgene.

As mRNA expression had not been previously assessed in *Hip14−/−* mice, we investigated transcript levels in WT, *Hip14−/−*, and BAC mice. A murine *Hip14−/−* specific intron-spanning primer was designed to anneal downstream (exon 14–15) of the gene trap used to create the *Hip14−/−* mice, which lies in intron 5. Murine *Hip14* transcripts were significantly reduced in *Hip14*−/− compared to WT littermates, but still detectable in *Hip14*−/− cortex. This was not significantly affected by the presence of the human *HIP14* BAC in BAC mice (Figure 1b).

### The human *HIP14* transgene generates a modest level of human HIP14 protein expression

We confirmed protein expression of HIP14 from the transgene using a previously described HIP14 antibody [18], which detects both mouse and human HIP14; thus, the band detected represents total HIP14 protein. A faint band visible in *Hip14−/−* lanes was confirmed to be non-specific, as it is not eliminated upon peptide competition assay using a >500 molar excess of the peptide used to generate the antibody (Figure S2). The human *HIP14* transgene is expressed at 36% and 35% of WT levels in striatum (Figure 1c) and cortex (Figure 1d) respectively. Protein expression was normalized to a beta tubulin loading control, and values are shown relative to wildtype levels. HIP14 levels were significantly increased in BAC mice relative to *Hip14*−/− in both striatum (p = 0.0091 normalized to WT, p = 0.0083 as raw values) and in cortex (p = 0.0016 normalized to WT, p = 0.019 as raw values).

### Human *HIP14* compensates for the neuropathological deficits in *Hip14−/−* mice

Mice lacking murine *Hip14* demonstrate neuropathological deficits, including a 17% loss in striatal volume by embryonic day E17.5, and an accompanying reduction in striatal neuron count [23]. In order to assess whether the protein expressed from the human *HIP14* BAC is functional, we assessed a series of similar neuropathological endpoints in *Hip14−/−* mice carrying the human *HIP14* BAC transgene, as compared to wildtype and *Hip14−/−* littermates.

Brain weight is significantly decreased in *Hip14*−/− mice throughout their lifespan [23]. In the current study, whole brain weight and cerebellum are significantly decreased at 1 month in *Hip14−/−* mice relative to WT; BAC mice are not significantly different from WT in whole brain, cerebellum, or forebrain weight (Figures 2a–c). One-way ANOVA analysis shows significant rescue in cerebellum (Figure 2b) and a trend to restoration of WT values in whole brain (Figure 2a) and forebrain (Figure 2c). Similar observations are seen at 3 months (data not shown).

Similar to previous findings [23], *Hip14−/−* mice demonstrate a 15.7% reduction in striatal volume at 1 month. This is fully rescued to wildtype levels in *Hip14−/−* carrying the BAC transgene (Figure 2d). Striatal neuron count is reduced by 14.3% at 1 month in *Hip14−/−* mice and this is also significantly rescued to WT values in BAC (Figure 2e). Finally, an 11.9% reduction of cortical volume in *Hip14−/−* at 1 month is rescued in the presence of human HIP14 (Figure 2f).

### Human HIP14 restores levels of DARPP32 and enkephalin in *Hip14−/−* MSNs

The striatal neuron populations affected in HD consist of GABAergic projection neurons and parvalbuminergic interneurons, while other neuron populations in the striatum remain relatively spared. The majority (95%) of striatal neurons consist of projection neurons; therefore, the striatal atrophy and cell loss observed in HD is largely due to loss of striatal GABAergic MSNs, cells that express high levels of DARPP-32 [37]. These MSNs are subdivided into two types: those expressing enkephalin and dopamine D2 receptors, and those expressing substance-P and dopamine D1 receptors. Levels of enkephalin and DARPP-32 are affected early in HD, whereas substance P remains largely unchanged until later in the disease [37]. As in HD patients, *Hip14−/−* mice demonstrate reduced levels of DARPP-32 and enkephalin in the striatum, while levels of substance-P are unchanged [23]. Similar to previous findings, striatal levels of DARPP-32 were reduced by ∼28% in *Hip14−/−* mice as compared to wildtype, and this was restored to wildtype levels in *Hip14−/−* expressing the human *HIP14* BAC (Figure 3a). Enkephalin levels were reduced by ∼56% compared to wildtype, while the BAC mice were similar to wildtype (Figure 3b). Striatal levels of substance-P were similar for all genotypes (Figure 3c). This data demonstrates that BAC-derived human *HIP14* compensates for the neurochemical deficits observed in the *Hip14−/−* mice.

### Motor coordination impairments & altered locomotor activity in the *Hip14−/−* mouse is restored to normal by human HIP14

*Hip14−/−* mice demonstrate deficits in motor coordination as early as 3 months of age [23]. We therefore assessed mice for performance on an accelerating rotarod at 3 and 6 months of age. Repeated measures ANOVA reveals a significant effect of genotype (Figure 4a). BAC mice performed consistently better than *Hip14−/−* littermates, remaining on the rotarod apparatus for a longer time before falling. BAC performance was also superior to WT littermates, and this was more apparent at later trials. A non-significant trend of WT performance superior to *Hip14*−/− is apparent. The consistently superior performance in BAC mice suggests that human HIP14 BAC can completely compensate for motor coordination deficits seen in *Hip14−/−* mice.

*Hip14−/−* mice are hyperactive in various measures of dark-phase assessment of spontaneous activity, similar to the observations of hyperactivity in young YAC128 mice [24], [25]. Spontanous activity measures were assessed at 3 and 6 months of age (Figure 4b–j). The most robust changes were observed in stereotypic time (Figure 4c) and counts (Figure 4f), where repeated measures ANOVA revealed a significant effect of genotype. Post-hoc Bonferroni testing revealed an increase in both measures in *Hip14*−/− vs. WT, and a robust rescue in BAC mice. A significant effect of genotype was also observed in ambulatory time (Figure 4b) and counts (Figure 4e), distance traveled (Figure 4d), time resting (Figure 4g), and ambulatory episodes (Figure 4j), where *Hip14*−/− mice displayed hyperactivity and a normalization to wildtype values in BAC was observed. In summary, human *HIP14* compensates for the behavioral deficits observed in mice lacking murine *Hip14*.

### Palmitoylation deficits of HIP14 substrates in *Hip14−/−* mice are normalized to wildtype levels by human HIP14

HIP14 functions as a PAT for a number of critical neuronal proteins, including PSD-95 and SNAP-25 among others [19]. Palmitoylation of these neuronal proteins is decreased in the *Hip14−/−* mouse model, and the defect in palmitoylation may underlie defects in trafficking observed in HD [23]. We sought to confirm that the functional enzyme activity of BAC-derived human HIP14 is intact, and to assess whether the human protein product can compensate for the loss of murine HIP14. Similar to previous findings, we observed reduced palmitoylation of PSD-95 in the *Hip14−/−* mice (Figure 5a). Palmitoylation of SNAP-25 was similarly reduced in the *Hip14−/−* mice, and returned to wildtype levels in the BAC mouse (Figure 5b).

### The reduction in body weight seen in *Hip14−/−* mice is only partially rescued by human HIP14

One of the features of HD in human patients is a progressive loss of weight [15], and *Hip14−/−* mice fail to gain weight [23]. We therefore assessed whether BAC-derived human HIP14 can compensate for this deficit. *Hip14*−/− mice aged 3 months demonstrate reduced body weight relative to WT, and this is partially rescued by human HIP14 in BAC mice in both genders (Figure 6). This pattern is more apparent by 6 months of age in both females (*Hip14−/−* 82.5% and BAC 87.6% of WT) and males (*Hip14−/−* 83.5% and BAC 90.8% of WT). Weekly measurements in a small subset of mice revealed that a similar pattern of partial rescue in body weight is present at early as 2 weeks of age (data not shown).

## Discussion

In this study we have demonstrated that human HIP14 can compensate for the *Hip14−/−* phenotype in functional measures of neuropathology, behavior, and PAT enzyme function. The similarities in phenotype between HD and the *Hip14−/−* mice highlighted a potentially important role for HIP14 in the pathogenesis of HD. However, it remained to be conclusively demonstrated that these phenotypes are the result of loss of HIP14 itself, and not, for example, a spontaneous mutation or a by-product of unintended mutagenesis events occurring in the generation of the *Hip14−/−* mouse model [38]. We created humanized HIP14 BAC transgenic mice by crossing human HIP14 BAC mice to mice lacking murine *Hip14*.

qRT-PCR experiments demonstrate that the transgene is expressed in BAC mice, but entirely absent in WT and *Hip14−/−* mice. However, the murine *Hip14* transcript is detectable in the *Hip14−/−* and BAC mice. This is not without precedent; similar detection of mRNA transcripts in gene-trapped mouse models has been previously reported [39]–[43]. It is possible that alternative splicing may allow excision of the gene trap vector, resulting in a low level of expression of the WT transcript. However, whether murine HIP14 is generated from the deleted transcript in the *Hip14−/−* mice was unclear. To determine if HIP14 protein is completely absent in the *Hip14−/−* mice, and to determine the level of HIP14 overexpression from the human BAC, we performed western immunoblotting.

Western blot analysis in cortex and striatum demonstrates loss of HIP14 in *Hip14−/−* mice, and expression at ∼35% of wildtype levels in BAC mice. Therefore, we have confirmed that human HIP14 is expressed from the BAC transgene. A faint band appeared in the *Hip14−/−* mice. This band appears to be non-specific, as it remains after the antibody was inactivated with a peptide competition assay (Figure S2), suggesting that any *Hip14* mRNA generated does not generate HIP14 protein.

As human and mouse HIP14 protein are highly similar (98% of amino acids identical), we anticipated that the human transgene would compensate for the loss of its murine ortholog. Previous studies demonstrated rescue of the murine huntingtin (*Hdh*−/−) knockout phenotype by human HTT despite only 90% conserved amino acid identity [29], [44]. In addition, previous findings showed that human HIP14 is able to partially compensate for the endocytosis defect and temperature sensitive lethality resulting from loss of Akr1p, a yeast ortholog of *HIP14* [18]. As anticipated, BAC-derived human HIP14 was capable of restoring a normal phenotype in neuropathological, behavioral, and functional enzymatic measures in mice lacking murine *Hip14*. Thus, our findings suggest that human HIP14 undergoes the necessary post-translational modifications and protein interactions necessary to perform these functions in the mouse cellular context, and can compensate for the loss of its murine ortholog in most measures assessed. Because neuropathological deficits in the *Hip14−/−* mouse appear during prenatal development by embryonic day E17.5, these findings furthermore suggest that the human *HIP14* BAC transgene is appropriately expressed in prenatal development.

As part of our study, we sought to assess what levels of HIP14 expression are sufficient to rescue the *Hip14−/−* phenotype. Surprisingly, the human *HIP14* transgene is expressed at only ∼35% of endogenous levels in cortex and striatum of mice lacking murine *Hip14*. Despite this relatively low level of expression, the neuropathological, behavioral, and biochemical measures of HIP14 assessed in this study are all fully rescued.

It is interesting to note previous investigations of another DHHC PAT null mouse model, *Zdhhc8*−/−, which revealed similar deficits in both heterozygotes (*Zdhhc8*+/−) and homozygotes (*Zdhhc8*−/−) relative to WT littermates [11]. For example, the density of glutamatergic synaptic contacts was decreased to a similar extent in both *Zdhhc8*+/− and *Zdhhc8*−/− mice. In addition, the number of mushroom spines, number of dendritic branch points, and number of primary dendrites were also affected in both genotypes [11]. In contrast, neuropathological assessments of *Hip14+/−* mice at 12 months revealed no loss in striatal volume, an endpoint showing the greatest changes in *Hip14*−/− mice as early as E17.5 [23]. As *Hip14*+/− mice express only 50% of endogenous HIP14, this data is in agreement with the findings of the current study; namely, that only a fraction of endogenous HIP14 expression levels are sufficient to prevent the neuropathological changes observed in *Hip14−/−* mice.

Many studies report partial rescue of murine knockout phenotypes by the human ortholog transgene. For example, human *NR2E1* rescues a murine *Nr2e1* null phenotype in all aspects except for retinal vessel number, which appeared to be partially rescued but still significantly different from wildtype mice [33]. Another study demonstrated the ability of a human *CFTR* YAC transgene to partially rescue the murine knockout phenotype [45].

While most major endpoints in the *Hip14−/−* mice were restored to wildtype levels in the presence of the human HIP14 transgene, the reduced body weight observed in the *Hip14−/−* mice was only partially restored to wildtype levels. The incomplete rescue of body weight in the humanized mice may occur for a number of reasons. Firstly, as observed for retinal vessel number in a similar study on *Nr2e1* null mice [33], maintenance of some physiological features may be more sensitive to gene dosage than the other endpoints assessed in this study. A higher level of expression (>35% of endogenous) may be required to restore body weight to wildtype levels. Alternatively, the role of HIP14 in the periphery may require a regulatory cis-element that is absent in the human BAC transgene used in this study. Finally, some functions of HIP14 may require an interaction between transcriptional machinery and DNA regulatory elements that is incompatible between the former in mice and the latter in humans.

### Conclusions

In summary, we have generated the first transgenic mouse model for *HIP14*, and demonstrate intact palmitoyl-transferease activity in a transgenic DHHC PAT model. Humanized mice for HIP14 were able to rescue defects in neuropathology, behavior, and HIP14 PAT activity, and partially restored body weight to wildtype levels. We report that human HIP14 compensates for the key features resulting from loss of the murine ortholog at an expression level equivalent to only ∼1/3 that of endogenous murine HIP14, which is sufficient for full rescue of most phenotypes.

This latter finding carries important therapeutic implications. We have previously shown that HIP14 is dysfunctional and displays impaired PAT activity in the brain of the YAC128 mouse model of HD despite normal levels of HIP14 protein [23]. The current study suggests that therapeutic interventions in human HD patients could be directed toward approaches to increase HIP14 activity, and that rescue of some phenotypes in YAC128 mice may be achieved with less than wildtype levels of functional HIP14.

## Supporting Information

Figure S1 **Creation of a** ***HIP14*** **BAC transgenic mouse.** **a.** Schematic of genomic DNA included in human *HIP14* BAC RP11-463M12, which includes ∼84 kb of upstream and ∼6 kb downstream regulatory sequence, excluding other intact genes or clearly defined promoter sequences. Numbers indicate location of seven primer pairs used to ascertain founders, listed in Table 1. **b.** PCR genotyping confirmation of tail DNA from FVB mice generated from microinjections with a human *HIP14* BAC. Eleven mice tested positive for the transgene, of which 9 were positive for all 7 primer sets assessed. Figure shows results for primer pair 2 (Table 1). BAC = *HIP14* BAC (5 ng), S = FVB WT genomic DNA spiked with *HIP14* BAC at 1-copy number (200 ng), WT = FVB WT genomic DNA, H20 = ddH_2_O, * = these mice not positive on all primer sets. **c.** qPCR assessment of relative transgene genomic copy number in *HIP14* BAC founder mice. *HIP14* BAC-spiked FVB genomic DNA was run in a standard curve for estimation of BAC copy number on the same plate as genomic tail DNA from each *HIP14* BAC founder mouse. Relative quantitation was calculated relative to the standard curve one-copy equivalent. Each sample was loaded in triplicate, and the plate run in duplicate. Error bars indicate the variation between the two plates. The highest BAC copy number was detected in lines HB2, HB4, and HB6. HB5F1 and HB7F1 are tail DNA from F1 offspring of founders HB5 and HB7, respectively, run on the same plate. **d.** qPCR assessment transgene copy number in the *HIP14* BAC F1 offspring reveals a pattern consistent with that observed in founders (n = 6). Copy number estimates of F1 were calculated relative to HB7.
(TIF)

Figure S2 **Peptide Competition Assay on PEP1 HD82 antibody for HIP14.** Identical WT and *Hip14−/−* samples of (**a**) Cortex and (**b**) Striatum lysate were run in duplicate on the same gel on SDS-PAGE gels and transferred to PVDF membrane. After blocking, membranes were cut in half and subsequently processed in parallel. One half of each membrane was incubated in PEP1 primary antibody according to the standard protocol (control). The remaining half of the membrane was inciubated with PEP1 primary antibody solution that had been pre-incubated with a >500 molar excess of the peptide used to generate the antibody. Subsequently, both membranes were washed and incubated with secondary antibody according to the standard protocol described in Materials and Methods. Beta tubulin was probed as a loading control. Incubation with peptide-competed primary antibody enables identification of non-specific bands. The bands that disappear upon peptide-competition are specifically recognized by the antibody; those that remain are non-specific. Notably, the faint band apparent in *Hip14−/−* samples remains in the peptide-competed membrane, indicating that this is a non-specific band.
(TIF)
