# Supplementary figures and images for: Low Levels of Human HIP14 Are Sufficient to Rescue Neuropathological, Behavioural, and Enzymatic Defects Due to Loss of Murine HIP14 in Hip14−/− Mice
Source: PLoS One. 2012 May 23;7(5):e36315. doi: 10.1371/journal.pone.0036315 (PMC3359340; doi:10.1371/journal.pone.0036315)

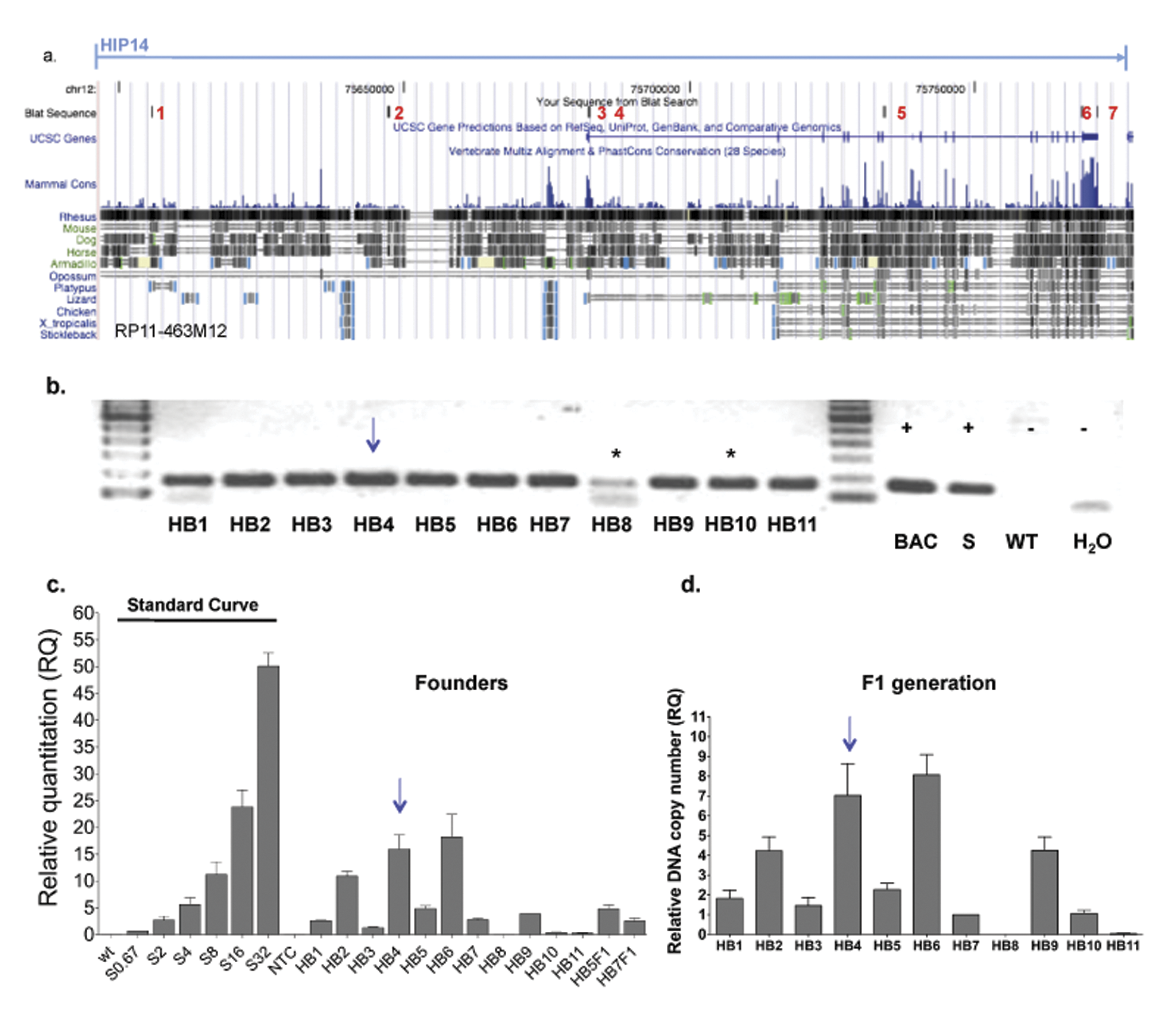

Supplement: Figure S1 — Creation of a HIP14 BAC transgenic mouse. a. Schematic of genomic DNA included in human HIP14 BAC RP11-463M12, which includes ∼84 kb of upstream and ∼6 kb downstream regulatory sequence, excluding other intact genes or clearly defined promoter sequences. Numbers indicate location of seven primer pairs used to ascertain founders, listed in Table 1. b. PCR genotyping confirmation of tail DNA from FVB mice generated from microinjections with a human HIP14 BAC. Eleven mice tested positive for the transgene, of which 9 were positive for all 7 primer sets assessed. Figure shows results for primer pair 2 (Table 1). BAC = HIP14 BAC (5 ng), S = FVB WT genomic DNA spiked with HIP14 BAC at 1-copy number (200 ng), WT = FVB WT genomic DNA, H20 = ddH2O, * = these mice not positive on all primer sets. c. qPCR assessment of relative transgene genomic copy number in HIP14 BAC founder mice. HIP14 BAC-spiked FVB genomic DNA was run in a standard curve for estimation of BAC copy number on the same plate as genomic tail DNA from each HIP14 BAC founder mouse. Relative quantitation was calculated relative to the standard curve one-copy equivalent. Each sample was loaded in triplicate, and the plate run in duplicate. Error bars indicate the variation between the two plates. The highest BAC copy number was detected in lines HB2, HB4, and HB6. HB5F1 and HB7F1 are tail DNA from F1 offspring of founders HB5 and HB7, respectively, run on the same plate. d. qPCR assessment transgene copy number in the HIP14 BAC F1 offspring reveals a pattern consistent with that observed in founders (n = 6). Copy number estimates of F1 were calculated relative to HB7. (TIF) [file pone.0036315.s001.tif]

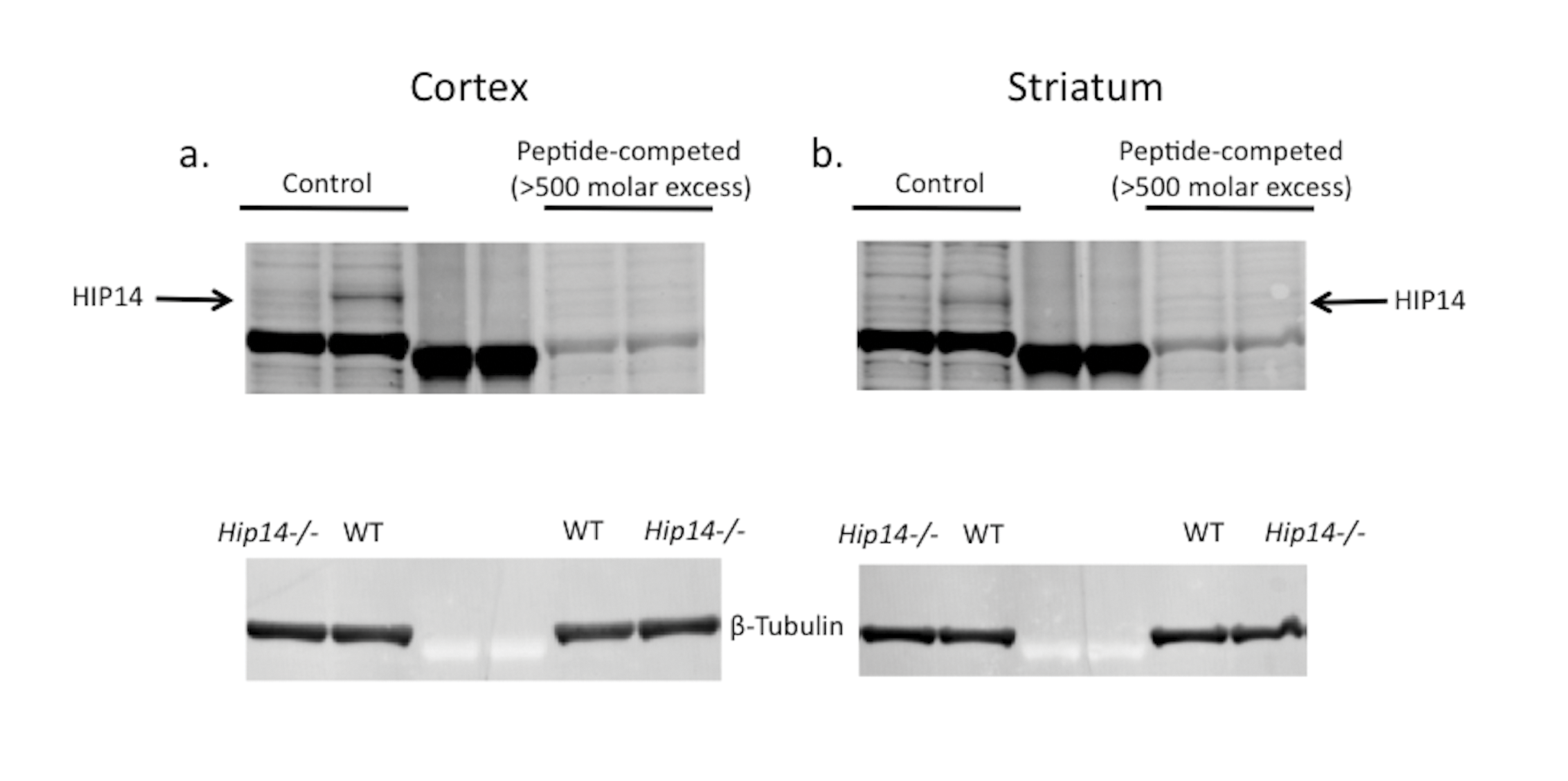

Supplement: Figure S2 — Peptide Competition Assay on PEP1 HD82 antibody for HIP14. Identical WT and Hip14−/− samples of (a) Cortex and (b) Striatum lysate were run in duplicate on the same gel on SDS-PAGE gels and transferred to PVDF membrane. After blocking, membranes were cut in half and subsequently processed in parallel. One half of each membrane was incubated in PEP1 primary antibody according to the standard protocol (control). The remaining half of the membrane was inciubated with PEP1 primary antibody solution that had been pre-incubated with a >500 molar excess of the peptide used to generate the antibody. Subsequently, both membranes were washed and incubated with secondary antibody according to the standard protocol described in Materials and Methods. Beta tubulin was probed as a loading control. Incubation with peptide-competed primary antibody enables identification of non-specific bands. The bands that disappear upon peptide-competition are specifically recognized by the antibody; those that remain are non-specific. Notably, the faint band apparent in Hip14−/− samples remains in the peptide-competed membrane, indicating that this is a non-specific band. (TIF) [file pone.0036315.s002.tif]
